# Amazonian freshwater habitats experiencing environmental and socioeconomic threats affecting subsistence fisheries

**DOI:** 10.1007/s13280-014-0610-z

**Published:** 2015-01-09

**Authors:** Cleber J. R. Alho, Roberto E. Reis, Pedro P. U. Aquino

**Affiliations:** 1 Programa de Pós-Graduação em Meio Ambiente, Universidade Anhanguera-Uniderp, Rua Alexandre Herculano, 1400 – Jardim Veraneio, Campo Grande, MS 79037-280 Brazil; 2 Laboratório de Sistemática de Vertebrados, Pontifícia Universidade Católica do Rio Grande do Sul, Pontifícia Universidade Católica do Rio Grande do Sul, Av. Ipiranga, 6681, Prédio 40, sala 151, Porto Alegre, RS 90619-900 Brazil; 3 Departamento de Zoologia, Universidade de Brasília, Campus Universitário – Asa Norte, Brasília, DF 70910-900 Brazil

**Keywords:** Amazon basin, Biodiversity, Fishery resources, Freshwater habitats, Environmental and socioeconomic threats, Seasonal flooding

## Abstract

Matching the trend seen among the major large rivers of the globe, the Amazon River and its tributaries are facing aquatic ecosystem disruption that is affecting freshwater habitats and their associated biodiversity, including trends for decline in fishery resources. The Amazon’s aquatic ecosystems, linked natural resources, and human communities that depend on them are increasingly at risk from a number of identified threats, including expansion of agriculture; cattle pastures; infrastructure such as hydroelectric dams, logging, mining; and overfishing. The forest, which regulates the hydrological pulse, guaranteeing the distribution of rainfall and stabilizing seasonal flooding, has been affected by deforestation. Flooding dynamics of the Amazon Rivers are a major factor in regulating the intensity and timing of aquatic organisms. This study’s objective was to identify threats to the integrity of freshwater ecosystems, and to seek instruments for conservation and sustainable use, taking principally fish diversity and fisheries as factors for analysis.

## Introduction

Human uses of natural resources have affected the freshwater ecosystems and the surrounding terrestrial environment during the recent decades of land use along all the large rivers on the planet. There has been worldwide concern over aquatic ecosystems, with freshwater biodiversity being the overriding conservation priority. River fisheries experts, meeting at the Symposium on the Management of Large Rivers for Fisheries, held in *Phnom* *Penh*, *Cambodia* in 2003, expressed alarm that freshwater fish stocks have declined in many of the world’s largest rivers (Dudgeon et al. 2006). Further evidence of this concern was the initiative of the United Nations General Assembly, which proclaimed the period from 2005 to 2015 as the International Decade for Action—”Water for Life.” The resolution calls for a greater focus on water issues to achieve freshwater conservation. The document “Living Planet Report 2014—Species and spaces, people and places” (http://wwf.panda.org/about_our_earth/all_publications/living_planet_report/) organized by WWF International, Zoological Society of London, Global Footprint Network, and Water Footprint Network, points out that although human beings are a product of the natural world, we have become the dominant force that shapes ecological and biophysical systems. In doing so, we are not only threatening our health, prosperity, and well-being, but our very future. Human well-being depends on natural resources such as water and fish, as well as ecosystem services.

Furthermore, biodiversity contributes to safeguarding subsistence use for traditional human communities (Millennium Ecosystem Assessment 2005). The document of the Intergovernmental Panel on Climate Change (IPCC) held in *Yokohama*, *Japan*, March 25–29, 2014, stated that a large fraction of both terrestrial and freshwater species face increased extinction risk under projected climate change during and beyond the twenty-first century, especially as climate change interacts with other stressors, such as habitat modification, over-exploitation, pollution, and invasive species (IPCC 2014).

From a biodiversity perspective, the Amazon basin is unequaled, with its aquatic habitats that are home to the world’s richest assemblages of freshwater flora and fauna, including reptiles (*Caiman* *crocodilus*, *Melanosuchus* *niger* among other crocodiles, *Podocnemis* *expansa*, *P.* *unifilis*, *P.* *sextuberculata,* and other turtles); amphibians; aquatic habitat-dependent birds, with good indicators of habitat quality (*Harpia* *harpyja*, *Morphnus* *guianensis*, *Neomorphus* *squamiger*, *Penelope pileata*, *Simoxenops ucayalae*, *Synallaxis cherriei*); mammals (otters such as *Pteronura brasiliensis* and *Lontra longicaudis*; river dolphins such as *Inia geoffrensis and Sotalia fluviatilis*, and manatee *Trichechus inunguis*); and more than 2200 fish species (Albert and Reis 2011; Alho 2011).

Other mammal species life history of which is connected with water are marsupials such as *Chironectes minimus*, *Micoureus demerarae*, *Marmosops noctivagus*, and *Marmosa murina.* Wild rodents strongly linked to habitats close to water are the semiaquatic *Nectomys squamipes* and the “toró” arboreal bamboo rat *Dactylomys dactylinus* usually seen vocalizing on branches over the water of rivers and seasonal lakes. Large mammals also exhibit dependence on aquatic habitats, like the semiaquatic capybara *Hydrochoerus hydrochaeris* and the tapir *Tapirus terrestris.*

The number of Amazonian aquatic species is a clear underestimation, because a significant portion of biodiversity is yet to be discovered and described, particularly considering amphibians and fish. The magnitude of the region is unique: the Amazon basin covers an area of approximately 7 000 000 km^2^, of which about 58 % (4 100 000 km^2^) is located in Brazil. These species play a fundamental role in the aquatic ecosystems because of their high diversity, and the ecological and evolutionary patterns they exhibit. For example, the Amazonian freshwater fish is very old and has distinct historical origins. Publications like “Check List of the Freshwater Fishes of South and Central America” (Reis et al. 2003), and Böhlke et al. (1978) point out the steeply ascending curve of new species being described, suggesting that the Amazonian number may exceed 3200 species.

The Amazon freshwater ecosystems are packed with the highest diversities of fish to be found anywhere on the planet (Lévêque et al. 2008; Albert and Reis 2011). The traditional socioeconomic activities such as fishing and extraction of other live natural resources have been affected by the latest increase in human migration, caused by new possibilities of access to land or driven by new infrastructure projects, livestock, agriculture, mining, commercial fishing, or other motivations. More than 24 million people live in Brazilian Amazonia (Santos et al. 2014).

In comparison with the *Mekong* River in Southeast Asia, one of the largest inland rivers and fisheries in the world, the total diversity of fish species—estimated at 1000 species—is significantly lower than that of the Amazon—estimated at more than 2200 (Albert and Reis 2011). The *Mekong* basin has been subjected to hydrological alterations, overfishing, and environmental degradation, and its fishery has been declining considerably over the recent years (Baran and Myschowoda 2008; Dugan et al. 2010).

Taking water discharge as a parameter, the Amazon River discharges 219 000 m^3^/s, while another of the world’s large rivers, the *Congo* River in Africa, discharges 41 800 m^3^/s (Reis 2013). The Amazon River is the largest single source of freshwater runoff on Earth, representing some 15–20 % of global river flow (Salati and Vose 1984).

Amazonian biodiversity is not only a source of biological and scientific wonder and fascination. The wealth of biological resources found in the Amazon biome has proven to be of enormous benefit to human well-being (Alho 2012). From wood, foods, and beverages to medicines and industrial products derived, for example, from the rubber tree, the biological diversity of the Amazon forest has had a profound effect on the development of human society. Many of these biodiversity secrets have been revealed to Science by indigenous peoples, whose livelihoods and cultures depend on their environment and surrounding biodiversity.

The Amazonian biodiversity represents a critical socioeconomic resource for local people who have long relied on fish resources as their major source of food and income (Ruffino 2004, 2005, 2008; Batista et al. 2012). Most notably, three freshwater turtle species are ecologically and socioeconomically important in the Brazilian Amazon due to traditional human consumption: the giant turtle *Podocnemis expansa*, the yellow spotted turtle or “tracajá” *Podocnemis unifilis*, and the six-tubercled turtle or “pitiú” *Podocnemis sextuberculata* (Alho 1985, 2011).

The “várzea” floodplain appears as the first focus of settlement of the Amazon region, with evidence of the presence of lowland indigenous peoples for about 2000 years. These indigenous groups sought the environments of rivers and streams (as a source of food and water) to settle their villages and spread through the region. However, the hydrographic network of the region not only conditioned the process of occupation by indigenous tribes and, later, by settlers, but also guided the path which the regional economy would take (Madaleno 2011). The different levels of landscape transformation, throughout the historical human occupation, are synthesized from landscape diversification to simplification and suppression (Lui and Molina 2009).

The aim of this paper is to identify and evaluate environmental and socioeconomic threats to Amazonian aquatic ecosystems to pursue the conservation and sustainable use of its freshwater biodiversity, taking fish diversity and fisheries as factors for analysis. Two primary concerns are established: (1) the protection of natural aquatic habitats and (2) the relationship of biodiversity resources, mainly fish and river turtles, to local people’s welfare.

## Materials and methods

**Fig. 1:**
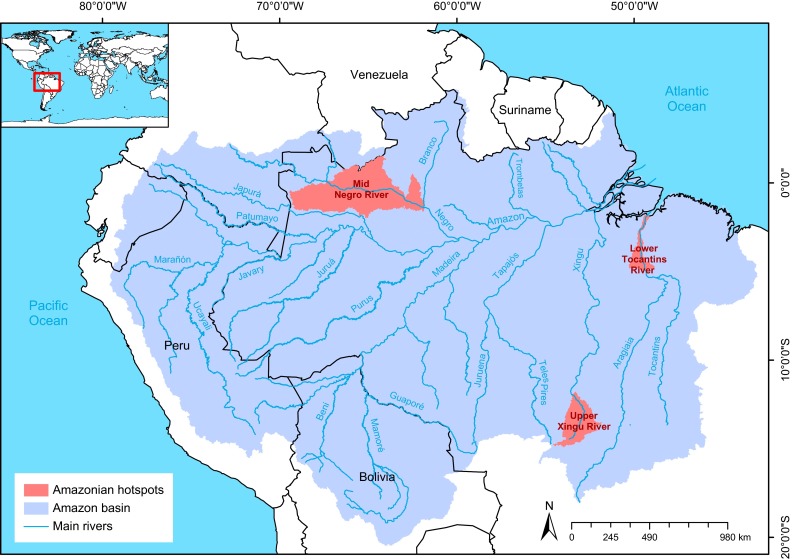
Study regions (upper Xingu River, Lower Tocantins River, and mid Negro River) Amazonian threatened areas, rich in aquatic biodiversity focus of the fieldwork

Field studies were carried out in three designated Amazonian threatened areas, rich in biodiversity, which are known as hotspots, providing a good representation of the different natural aquatic habitats: the Upper Xingu River, the Lower Tocantins River, and the Mid Negro River, highlighting the aquatic biodiversity, with emphasis on the diversity of fish and fisheries in their various forms in different kinds of aquatic habitats (Fig. 1). Ten days were spent working at each site, visiting different habitats, interviewing riverine inhabitants, fishermen and other fisheries professionals, and covering a sampling path of about 300 km in each hotspot. The interviews were performed to detect the riverine fishermen’s perception of the environmental threats and fishing conflicts under a qualitative or descriptive focus rather than a quantitative ones. The people targeted by the interviews were the leaders of the organized fishermen’s institutions and other actors in the fishing productive web, such as artisanal fishermen; the owners of ice boats who purchase the fish to transport to consumer centers and other intermediate traders; the leaders of the fishing colonies and cooperatives; and others. Among the interview topics were fishing arts and artifacts, including the mesh size of fishing nets; the use of underwater harpoons; period of closed fishing season and government financial support to fishermen during the closed season; the role of legislation and fishing regulation enforcement; conflicts between resident artisan fishermen and larger fishing boats; conflicts between riverine fishermen and sport fishing activities, as well as perceptions of different fishing stressors such as riparian deforestation; advance of agricultural fields and cattle pastures; effects of infrastructure such as hydroelectric dams, roads, mining, boat traffic; and water pollutants. We conducted the interviews to assess the attitudes and perceptions of fishers and other actors toward the environmental threats and fishing conflicts. Surveys were based on informal group discussions conducted with fishers and other respondents. Participants within the fishing areas were chosen opportunistically. We surveyed fishers belonging to both traditional subsistence and commercial categories. We qualitatively analyzed positive perceptions, oral histories, and convictions, related to fishing activities, documented in survey responses to open questions. We also examined the fish catch harvested across the study sites.

### Study sites

The Xingu River is a major tributary of the Amazon basin and drains the Brazilian Shield, along with the Tocantins, Araguaia, Tapajós, and part of the Madeira rivers. The Xingu River basin forms the Aquatic Ecoregion 322 of the freshwater ecoregions map of the world (Abell et al. 2008). In April 2013, 300 km were covered in a motorboat throughout the headwater region of the *Xingu* River, sampling the selected areas (Table 1).

**Table 1:** Study sites in the Upper Xingu River region informing geographic position and altitude of each site

Study sites	Latitude	Longitude	Altitude (m)
Headwater of the *Xingu* River	−12.880338°	−52.814270°	300
Jointure of *Culuene* and *Sete de Setembro* rivers	−12.924960°	−52.825969°	302
Upper *Culuene* River	−13.077444°	−52.885847°	310
Coronel *Vanick* River	−13.126701°	−52.689272°	304
Upper Coronel *Vanick* River	−13.237053°	−52.629633°	317
Upper *Sete de Setembro* River	−13.239636°	−52.551104°	311
Upper *Xingu* River	−14.045409°	−52.344515°	355

This region is an ecotone zone, transition between the savanna (Cerrado) biome of central Brazil and the Amazon basin. This study area was chosen for three main reasons: (1) the Xingu River represents the tributaries of the right-hand side of the Amazon, which have their headwaters on the Brazilian Shield; (2) it is an endemic region for fish species: out of 142 fish species recorded for the Xingu basin, 36 species are endemic (Albert and Reis 2011; Buckup et al. 2011), and most of this endemism occurs in the upper Xingu basin; and (3) this region has been under strong threat of deforestation.

The second selected study site was the Lower Tocantins River, inside ecoregion 324—Tocantins–Araguaia—(Abell et al. 2008). In early June 2013, during the fieldwork, approximately 300 km were covered along the Tocantins River, sampling the selected sites (Table 2).

**Table 2:** Study sites in the Lower Tocantins River region informing geographic position and altitude of each site

Study sites	Latitude	Longitude	Altitude (m)
*Baião* village	−2.792377°	−49.674500°	10
*Joana Peres* village	−3.016875°	−49.748470°	12
*Ituquara* village	−3.031125°	−49.646270°	13
*Vila Nova* village	−3.450295°	−49.608875°	13
*Moru* village	−3.558638°	−49.612157°	14
*Tucuruí* city	−3.756088°	−49.664309°	16
*Tucuruí* dam	−3.832021°	−49.643278°	70
*Porto do Onze*	−3.848169°	−49.680821°	70
*Porto Novo*	−4.414191°	−49.387704°	73
*Santa Rosa* village	−4.522811°	−49.408602°	73

The Lower Tocantins River was selected for field study, considering the following reasons: (1) it is a river strongly impacted by a huge hydroelectric dam and reservoir (Mérona et al. 2010); (2) it presents high endemism of fish species; and (3) it is an important Amazonian river, extending up to 2750 km in length.

Finally, the third study site selected was the Mid Negro River, included in ecoregions 314 and 315 (Abell et al. 2008). In late July 2013, 310 km along the Negro River were covered, throughout the selected sampling areas adjacent to the town of Barcelos (Table 3).

**Table 3:** Study sites in the Mid Negro River region informing geographic position and altitude of each site

Study sites	Latitude	Longitude	Altitude (m)
Fishing area 1	−0.856116°	−62.765446°	35
*Barcelos* city	−0.966978°	−62.928499°	30
*Bacabal* village	−0.483100°	−62.921512°	29
*Daracuá* village	−0.506514°	−63.214250°	27
*Ponta da Terra* village	−0.770953°	−63.142164°	25
*Aracá* River	−0.771188°	−62.937692°	28
*Cubá* Stream	−0.924075°	−62.917655°	29
*Mamulé* Stream	−0.808618°	−63.237575°	27
*Murumuru* Stream	−0.467140°	−62.928506°	39
Fishing area 2 (*Calibuque*)	−0.629722°	−62.868497°	33
Fishing area 3	−0.464315°	−63.154313°	28
Fishing area 4	−0.466774°	−62.936246°	40
*Demini* River	−0.414927°	−62.903415°	44
Fishing area 5	−0.833065°	−63.231232°	35
Fishing area 6 (*Zamula*)	−0.865862°	−62.774625°	32

The Negro River was chosen because of the following reasons: (1) it is entirely contained inside the lowland forested area of the Amazon basin, being the major black-water river on the left bank of the Amazon River, and it is the largest black-water river in the world; (2) its black water is poor in mineral nutrients but presents fish endemism; and (3) traditionally, the region of the Mid Negro River supports an extractive activity of ornamental aquarium fish with high socioeconomic relevance.

The identification, characterization, and evaluation of environmental threats to aquatic ecosystems are important tools in planning strategic conservation actions. These aspects of threats were therefore involved in finding ways to implement policies and environmental management, taking into account any particular environmental modifier that potentially threatens the Amazonian aquatic environment. Some of these threats to aquatic ecosystems are of a scientific and technical nature, such as those that influence the structure and function of ecosystems. Others are of a political and administrative nature, and can often be anticipated and avoided, such as the legal fishery standards that must be implemented and followed.

Identifying and qualifying the effect of the threat is somewhat subjective, since it involves not only the ecological focus, but also the socioeconomic point of view. Therefore, the environmental variable threat translates into the integration of physical and chemical components (soil, water quality, etc.), as well as the biotic component (flooded vegetation, aquatic biodiversity, etc.) and socioeconomic elements (fishing, hunting, capture of turtles and their eggs, extraction of other natural products, social organization of fishermen, etc.).

A wide range of environmental threats, detected during the fieldwork, interact with natural biological and physical components of the Amazon freshwater ecosystem to change biodiversity productivity and ecological community structure.

## Results

While conducting fieldwork in the three designated river basins (Upper Xingu River, Lower Tocantins River, and Mid Negro River) some environmental threats to the aquatic ecosystems and their biodiversity were identified (Table 4).

**Table 4:** Identification and description of environmental and socioeconomic threats in the three Amazonian river basins sampled during the fieldwork: Upper Xingu River, Lower Tocantins River, and Mid Negro

Threat	Description of threat	Upper *Xingu* River	Lower *Tocantins* River	Mid *Negro* River
Alteration and loss of fish habitats due to deforestation of riparian vegetation	Habitat conversion of riparian communities, from expansion of agriculture, cattle ranching and urbanization in floodplains	Intensively verified	Moderately verified	Not yet relevant
Effect of reduced river flow due to deforestation of headwater areas	Changes in upland areas (deforestation, expanding cattle ranching, urbanization) resulting indirectly in greater sediment loads and contaminants such as fertilizers and pesticide from run-off	Observed conversion of natural vegetation into huge areas of soybean crop fields	Moderately verified	Not yet relevant
Effect of environmental contaminants on the water quality	Direct contamination of rivers from increased dumping of organic and solid waste into rivers from expanding urban areas and from activities such as intensive agriculture and mining	Potential contamination of waters from agricultural fertilizers and pesticides	Urban sewage pollutants	Urban sewage pollutants
Effect of hydroelectric reservoirs on diversity and fish communities	Transformation of a lotic environment into a lake eradicating or reducing populations of rheophilic fish species, and providing favorable conditions for lentic species to proliferate	Moderate and specifically located effect from small hydroelectric plants	*Tucuruí* dam and plant operation: overall drastic effects on natural habitats and fish communities	Not relevant
Effect of infrastructure on diversity and fish communities	Changes in hydrological regimes through construction of infrastructure such as roads, ports and navigation channels	Moderate impact	Severe impact	Not relevant
Threats to turtles and freshwater mammals	Illegal hunting and commerce of wildlife	Moderate impact in the upper region but very severe in the lower portion (*Belo Monte* region)	Severe impact	Moderate impact
Effects of fishing conflicts	Detected growing number of conflicts among natural resource users	Moderate conflict between sport and subsistence fishing	Severe conflict among commercial fishing interests	Moderate conflict between ornamental and sport fishing
Decline of ornamental aquarium fishing	Capture of ornamental fish species for aquarium international commerce	Not relevant in the upper region but important in the lower area of Volta Grande do *Xingu*	Not relevant here	Negative socioeconomic effect due to decline in demand; fishermen are now starting to seek their livelihoods in other activities, such as agriculture
Effects of deficient implementation of fishing regulation	Lack of organizational and institutional capacity to deal with fishing in a participatory and integrated manner	Sport fishing observed here to be organized to take advantage of immediate income of fishing opportunity	Several fishing colonies exist but conflicts are still severe due to the lack of fishing regulation and implementation	Weakness of organization due to present decline in ornamental fishing
Effects of deficient dialogue between participant actors of fishing social organizations	Social organizations play a significant role in enabling fishery management to ensure sustainability	Strong organization among owners of sport fishing accommodation but low concern for sustainability	Conflicts among subsistence, commercial and sport fishing	Drastic decline in dialogue due to decline in demand for ornamental fishing
Effects of overfishing	Overexploitation of fishery resources is observed when fish stocks are suppressed to a level where fishing in a given region is no longer sustainable, that is, there is a need to increase fishing effort and stocks are not replenished naturally	Competition between sport fishing and artisanal subsistence fishing. Smaller fish sizes.	Observed strong pressure on fishing stocks to meet demand	Sport fishing in increasing demand and ornamental fishing in decline
Effects of global climate change on aquatic environments	The published literature indicates that it appears highly possible that the predicted climate change over the next decades may well cause additional damage to Amazon aquatic ecosystems	Regionally subject to drying change due to intense and extended deforestation	Regionally subject to change due to drastic environmental change—*Tucuruí* dam	Forest still well preserved

The identified environmental and socioeconomic threats imply that in addition to the decline in quantity and quality of fishery resources, based mainly on field observations, there is also a reduction in the size of fish being captured, and this trend has a strong linkage with regional human demographic growth in Amazonia. As a consequence, there is an increasing demand for fish and other natural resources, and conflicts have arisen between commercial and subsistence fisheries.

### The Upper Xingu River: Sport fishing and the threat of soybean fields

The Xingu basin is the fourth largest watershed of the Amazon, comprising seven percent of the region. The higher altitudes of the headwaters are located in the state of Mato Grosso at 800 m elevation. The Xingu River runs on crystalline rocks, carrying a low amount of sediments, with clear water. The waters start to rise in September–October, reaching the maximum level in March–April.

The upper Xingu region has been under strong pressure of deforestation. Local inspection followed by analysis of satellite images has shown immense areas adjacent to visited rivers (Culuene, Sete de Setembro, Xingu and others) converted into arable fields and cattle pasture, as observed during the fieldwork. The advance of agriculture has been driven by government programs which encouraged crops in the region. In this area, the Brazilian Savanna biome had already been highly converted by 2008, according to the deforestation monitoring program PRODES (INPE 2008). Local observations show that crop fields have now advanced to the edge of the rivers, altering or eliminating the riparian forest. This radical deforestation violates Brazilian legislation (Brazilian Forest Code, Law 12 651/2012), which protects a strip of the riparian forest on both sides of the river as an area of permanent preservation for large rivers. The seasonally inundated riparian forest constitutes important feeding and reproductive habitats for fish. In addition, water contamination through the intensive use of fertilizers and pesticides, which are usually spread by airplane on the crop fields, potentially affects the freshwater wildlife.

The region also attracts sport fishing, and several fishing resorts were observed along the visited rivers. Local people interviewed reported a decline in fish quantity during the last 10–15 years. They also claim that more recently the size of fish collected by sport fishing has decreased.

### The Lower Tocantins River: Infrastructure plants and change in the fish ecological community

The Tocantins River is a major tributary of the Amazon basin and drains, for the most part, from the Brazilian Shield. The catchment basin of the Tocantins River drains an area of 767 000 km^2^ (Mérona et al. 2010). The last portion of these environments is now submerged by the Tucuruí hydroelectric reservoir.

The Tocantins basin is the third largest sub-basin of the Amazon River, with an average annual discharge of 11 000 m^3^ per second. Its waters are clear and nutrient-poor due to draining away the crystalline formations of the Brazilian Shield. In this basin occurs a high endemism of fish species: “piranhas” such as *Serrasalmus geryi* and *S. eigenmanni*, and other species like “aracus” *Leporinus affinis* and *L. taeniatus*, the ray *Potamo trygonhenley,* and others (Mérona et al. 2010).

In 1984, the *Tucuruí* hydroelectric plant was built in the lower portion of the river. The dam created a 200 km-long reservoir with an area of 2875 km^2^ (Mérona et al. 2010). Within the upper dam, to the north of the Tocantins River basin, still freely connected to the rest of the Amazon basin, some fish species occur that are absent or rare in today’s portion under the influence of the dam. The Tucuruí dam has caused a decline in fish diversity living in the reservoir (Mérona et al. 2010). The change from a lotic environment in the reservoir eradicated or significantly reduced populations of the rheophilic fish species and provided the conditions for lentic species to proliferate, thereby altering the composition of the local fish community. Detritivorous, herbivorous and insectivorous fish decreased in abundance in the reservoir. At the same time predators, omnivorous and planktivorous species, were favored and increased in abundance (Mérona et al. 2010). Surveys at local fish markets during the fieldwork showed that some abundant commercial species caught in the reservoir are more adapted to a lentic environment, such as “mapará” *Hypophthalmus marginatus*, “tucunarés” *Cichla monoculus* and *Cichla* sp., and “corvina” *Plagioscion squamosissimus*.

Downstream from the dam, the decrease in abundance of fish species is mainly due to the regulation of river flow, which greatly disturbs the annual feeding and reproductive cycles of fish. The dam also interrupts migratory routes, since migratory fish cannot cross the barrier created by the dam, which has no fish ladders, to spawn upriver. Threats to fish and their aquatic habitats were identified during the fieldwork. Fishermen from fishing colonies were interviewed on the lower river region of Tucuruí: Colony Z-53 Breu Branco, Colony Z-43 Porto Novo Jacundá, both within the reservoir, and Colony Z-34 Baião downstream from the reservoir. Interviewees stated that there has been a significant decrease in fish abundance, both in the lake and downstream, especially in the last 10–15 years.

Local fishermen interviewed condemned the use of underwater harpoon fishing, a practice that has become commonplace in the last decade, as it allows for the capture of “tucunarés”—when adults feed little with restricted movement during the period in which they are defending spawning territories or taking care of offspring, and for this reason are not captured by the usual gear, like gillnets and hooks. The capture of these individuals displaying territorial reproductive behavior affects all offspring and thus has a great predatory effect. Most respondents complain of lack of legislation and fishing regulation enforcement, reporting that government officials rarely appear throughout the year or during the period of the closed fishing season. Because of this, interviewees stated that the closed season is not observed by most fishermen in the region.

According to the majority of the fishermen, however, the primary responsibility for the sharp decline in fish abundance after closing the Tucuruí dam is the daily downstream fluctuation of the water level and, especially, the weekly variation in river level as a function of the flow of water in turbines to attend different hours of energy production. This area was also visited during the fieldwork. The greatest demand for electricity in the early evening of each day, as well as lower demand by the industries on weekends, leads to a regulation of the amount of water passing through the turbines that is released into the river, causing a small increase in river level every night, and a reasonable decrease in level every weekend. These changes in water level disrupt the natural cycles of fish feeding and reproductive behavior, being especially destructive in the spawning season (October–March). Many of these areas covered during the fieldwork are shallow, and small variations in river level, such as those produced by the retention of water on the weekends, are sufficient to expose to the air and thus kill juvenile fish and eggs laid in the mass of vegetation.

The Tocantins River already has several hydropower plants in operation, and several others planned to be built. The existing threats here identified will then be intensified.

### The Mid Negro River: Buy a fish and save a tree

The Negro River is entirely contained in the Amazon sedimentary basin, being the largest tributary of the left bank of the Amazon River. Most of this basin is on a lowland floodplain, rising to about 100–250 m altitude in the west and down to about 50 m altitude near its mouth.

The black water of the Negro River is extremely low in mineral content. It is 2230 km long, with a catchment area of 696 000 km^2^, and an average flow of 28 400 m^3^ per second, which represents 14 % of the annual average flow of the Amazon basin (Filizola et al. 2010). It is estimated that an area of 30 000 km^2^ of the Negro basin is flooded seasonally between four and eight months of the year.

The current estimate of fish species richness in the Negro River basin exceeds 750 described species. Over 90 species are endemic to the basin, as well as some monotypic genera (*Tucanoichthys*, *Ptychocharax*, *Atopomesus*, *Leptobrycon*, *Niobichthys, and Stauroglanis*) currently found only in this basin (Petry and Hales 2013). An example of endemic species is the small catfish *Denticetopsis sauli* found only in the headwaters of the Negro River. The fish community composition differs from other parts of the Amazon basin primarily in its ecological constraints imposed by nutrient-poor black waters and low pH.

The iridescence of some species, such as the cardinal-tetra *Paracheirodon axelrodi*, may be an adaptive trait to live in the black water of the Negro River. Interesting ecological phenomena include large migrations of some fish species. The “jaraqui” (*Semaprochilodus insignis*), for example, migrates from the black waters of the Negro River to the white-water rivers to spawn. There are also unique assemblages of species in deposits of leaves and miniaturized habitats. Relictual species in this region include the arapaima *Arapaima gigas*, one of the largest freshwater fish in the world, of more than two meters in length, the “aruanã” *Osteoglossum bicirrhosum*, and the endemic black “aruanã” *O. ferreirai* (Petry and Hales 2013).

This region is distinguished by the large number of ornamental fish species captured to supply the international demand for ornamental aquarium fish. The interviewees revealed that there are over 100 commercially exploited aquarium species, and the small cardinal-tetra contributes over 80 % of individuals captured. To compare the local inspection to satellite images, it was confirmed that the forests of the mid Negro region are still very well preserved and, only recently, with the sharp decline in ornamental fishery, have areas begun to open up for subsistence agriculture. One human community visited, Daraquá, located in a flooded “igapó” area, had more than 20 families about 10 years ago, all relying exclusively on ornamental fishing. With the recent decline in the ornamental fishing trade, families were gradually abandoning the community and moving to land areas where other economic activities are possible. Therefore, in these new areas, a considerable portion of upland forest was removed to accommodate plantations of cassava, maize, and other crops. Currently, only a father and son from one family remain in Daraquá, where they rely on the capture of ornamental fish.

The “Projeto Piaba” (http://opefe.com/piaba.html) is a community-based interdisciplinary project established to understand the ecological and sociocultural systems of the *Negro* basin, in order to conserve and maintain the live ornamental fishery and other renewable resources at a commercially feasible and ecologically sustainable level. According to the interviewees, until around 10 or 12 years ago, the town of *Barcelos* had about 600 families relying on capture and commerce of ornamental fish. That figure has now fallen to less than ten percent of the previous number, and the main economic activities have become agriculture and guiding tourists for sport fishing, especially for “tucunarés.” Indeed, nowadays the sport fishing season, which occurs between August and February, is what drives the city, both socially and economically.

Ornamental fishery used to be the main economic activity of the city of Barcelos and other communities of the Mid Negro, having started in the mid-twentieth century. It is estimated that, at the time, about 20 million ornamental fish were exported every year from Barcelos. Different stakeholders interviewed informed us that during the last 10–15 years, the entire production chain of ornamental fish in the Mid Negro River had severely declined. The direct and immediate cause of this decline is a sharp drop in demand for ornamental fish. Indirect causes underlying decrease in demand, however, are less clear and have multiple origins. Apparently, competition from new and growing export markets for ornamental fish in Peru, Venezuela, and especially Colombia, charging even lower prices than Brazil, have increased difficulties for Brazilian fish exporters. In addition, several commonly exported fish species from the Negro River can now reproduce in captivity, in the USA and some Asian countries such as Malaysia and Singapore, at reduced costs. Also, the cardinal-tetra, the main species captured in the Negro River, is now under management control to reproduce in captivity in the USA and the Czech Republic, albeit on a small scale.

The decline in ornamental fishery is believed to have had a negative impact on regional forest conservation. For example, the “Piaba Project,” created by ichthyologist Dr. Labbish Chao, from the University of Amazonas at Manaus, implies that unlike mining, logging, agriculture, and other activities, ornamental fishing is not a destructive activity. This perception is very clearly expressed in the slogan of “Projeto Piaba”: buy a fish and save a tree.

## Discussion

The Amazon’s aquatic ecosystems linked to all biodiversity resources as well as human riverine communities, including in many places indigenous peoples who depend on natural resources, are increasingly at risk from the identified threats here described. Changes in fish species composition, alteration of the relative abundances of species and their source of food, and changes in reproduction and productivities of populations are some of the consequences of the environmental threats and unsustainable use of fishery resources by humans.

Natural changes are part of any aquatic ecosystem. However, present human demographic growth and land use in Amazonia, combined with rapid increases in fishery consumer demands, are bringing new pressures to damage fish resources and driving more profound changes, more rapidly than any natural impact has ever done.

The identified threats on the effects of reduced river flow due to deforestation and infrastructure (Table 4) affect feeding and reproductive strategies of fish. In fact, the seasonal dispersion of fish to perform feeding and reproduction strategies is one of the most striking behavioral complexes of the Amazonian ichthyological fauna. The majority of species move between the river and the adjacent flooded forest, including the well-known species such as “curimatã” *Prochilodus nigricans*, “jaraquis” *Semaprochilodus* spp., and “pacu” *Myleus* spp. (Batista et al. 2012). The floodplains support organic production of the aquatic ecosystem and are directly responsible for fisheries’ productivity, guaranteeing fishing yields and the maintenance of biodiversity. In addition to fish-feeding strategy as a function of the flooding regime, fish-reproductive strategy depends mainly on the flooded season, since the majority of Amazonian fish rely on inundated habitats to reproduce. The productivity of the aquatic ecosystem depends on two factors: (1) the amount of nutrients washed down by water from the headwaters, from the Andes, the Brazilian, and the Guyana Shields; and (2) from the extent of the floodplain (lowland flooded forest). Important representatives of fish in the seasonally flooded aquatic ecosystems are detritivorous species that feed on decaying plant debris, followed by species of herbivores, and frugivores.

Heterogeneous types of forested riparian vegetation cover about 30 % of the Amazon basin (Junk et al. 2014). The edges or banks of the rivers form a different complex of aquatic habitats, depending on the season of the hydrological cycle. Also the marginal lakes change according to the season of the year, since they have depressions or passages linking to the main course of the river.

Seasonal flooding is one of the key elements that control productivity and food webs. The flooded areas create new habitats and the movements of waters carry nutrients that supply the food webs. These seasonal forces favor productivity, promoting availability of new aquatic habitats to supply feeding and reproductive niches for several species. The biogeochemical cycle is an important process controlling the productivity of the aquatic ecosystem, combining physical and biological components of the natural system. While river dolphins, turtles and fish are able to respond promptly to the physical component of seasonal flooding, they are also integrated in the biological complex of competition and predation, as another component of trophic linkages.

During the beginning of the dry season, when resources are becoming scarce, many fish migrate into the river channels and change feeding strategy. Therefore, the feeding niches fluctuate depending on water flow. The evolutionary strategies of various fish species vary depending on the food supply: the predator piscivorous species such as the “tucunarés” (*Cichla* spp.) and the “pirarucu” *Arapaima giga*; the carnivores such as the “acará” *Geophagus altifrons* that feed on crustaceans, molluscs, bryozoans and insects; the herbivores that feed on aquatic macrophytes, such as “aracus” *Rhytiodus* spp.; the detritivorous species which are those that consume decaying organic matter, such as “jaraquis” *Semaprochilodus* spp.; and the omnivores that use various sources of food, such as “tambaqui” *Colossoma macropomum*.

There is a synchrony between fruit production in the inundated forest and the flooding season. Frugivore fish disperse to the flooded forest to feed, such as species of the genera *Colossoma*, *Bryconops*, *Tocantinsia*, *Leporinus*, *Tometes*, *Myleus* and *Triportheus*. In addition, these species play an important role in dispersing these forest seeds, contributing to the maintenance of the ecosystem. At low tide, the waters return to the main river channel, carrying decaying organic matter, which also contributes to the productivity of the aquatic ecosystem. Some fish species, such as *Myleus rhomboidalis*, change their feeding strategy depending on the season. During high water they feed on fruits in the flooded forest. During the dry season, they return to the river and feed on small prey such as invertebrates (Batista et al. 2012).

The ecological interactions among all the abiotic and biotic components of the freshwater ecosystem, including river types, flood regimes, distinct riparian forest, and many other elements of local biodiversity, are responsible for a complex mosaic of aquatic habitats. An important aspect to emphasize is that, depending on the specific water composition, the amount and quality of sediment and nutrients influence primary productivity, fish diversity, and community structure. These ecosystem components influence water visibility and electric conductivity, in consequence affecting the behavioral ability of fish to detect visual signals (characiform fish) or electric stimuli (gymnotiform fish) to swim, forage, escape from predators, and reproduce.

However, there is a group of sedentary fish species that are able to stay in the same habitat all year round, tolerating a low level of oxygen. This group includes “tucunarés” *Cichla* spp., “acarás” *Geophagus* spp., “aruanã” *Osteoglossum bicirrhosum*, “pirarucu” *Arapaima gigas*, various species of “piranhas” *Serrasalmus* spp., and *Pygocentrus* spp., among others (Fabré and Barthem 2005).

The Imazon Deforestation Alert System detected a deforestation area of 838 km^2^ in August and September 2014, in Amazonia, which represents an increase of 191 % in relation to 288 km^2^ of August–September 2013 (www.imazon.org.br/publication/forest-transparency/deforestation-report-for-the-brazilian-amazon-august-2014-sad). The impacts on the biogeochemical cycle, as consequences of clearing and burning of the Amazon forest, are being raised as the causes of extreme droughts in southeastern Brazil, with climate change on broader scales (Cavalcanti 2012). Flooding or droughts affect the population and several sectors of the economy.

The effect of hydroelectric dams has a strong influence on fish movement and community structure (Table 4). Some species that exhibit long-distance migration along rivers, swimming thousands of kilometers upriver to lay eggs, such as the catfish “piramutaba” *Brachyplatystoma vaillanti* and “dourada” *B. flavicans,* find the dam an obstacle in their migration route (Fabré and Barthem 2005). The environmental stimulus that triggers this behavior is a complex set of elements that include water level and turbidity as well as available seasonal habitat structure. Lotic environment of the reservoir reduces rheophilic fish species and favors lentic species such as the phytophagous strategists that filter plankton like the “maparás” *Hypophthalmus* spp., found in abundance in the Tucuruí reservoir, as has been observed in our field studies. The Lower Tocantins River suffers from change in the fish community ecology, since the Tucuruí dam has caused the decline in fish diversity living in this area.

Besides the Tucuruí dam, examined in this study, there has been increasing construction of large hydroelectric dams in the Amazon, such as Jirau and Santo Antônio on the Madeira River and Belo Monte on the Xingu River. In addition, there has been a proliferation of hydroelectric dams in the Andean Amazon, with potential ecological impacts in terms of river connectivity and forest loss (Finer and Jenkins 2012). There are plans for 151 new dams greater than 2 MW over the next 20 years, more than a 300 % increase. The reservoir formed in an area previously without that body of water now has an influence on the phenological rhythms of the surrounding vegetation as well as altering the seasonal phases of the hydrological cycle (flood phase, drying phase, drought, and flooding phase). The movements of fish, aquatic mammals, and freshwater turtles fit in with this hydrological cycle, in function of food availability and appropriate reproductive habitats (Alho 2011).

Our survey shows that hydrological disruptions, together with a fast growing human population and increase in deforestation, provide major challenges in the way fisheries resources are sustainably used in the region.

The threat to turtle species has an impact on biological as well as on socioeconomic components (Table 4). Traditionally, in the Amazon, there is consumption of turtle meat and eggs, mainly by traditional peoples, for whom this is an ingrained cultural habit. Besides fish, turtles are important inhabitants of aquatic ecosystems. During the nesting season, the Amazon turtle *Podocnemis expansa* migrates from lakes connected to the rivers to selected areas of river beaches to lay eggs (Alho and Pádua 1982). This movement is synchronized with the ebbing regime and lowest water.

The threat of the decline of ornamental aquarium fishing (Table 4) showed that the Mid Negro River area, like other regions in the Amazon, is distinguished by the large number of ornamental fish species captured to supply the international demand for ornamental aquarium fish, but recently it has suffered a decline in the ornamental fishing trade, and people have abandoned the area, looking for other sources of income. In the Negro River, there are also unique assemblages of species in deposits of leaves and miniaturized habitats.

The growing number of conflicts detected among natural resource users (Table 4) revealed disagreements between commercial and subsistence people. The fishers reported a decline in their potential catch and livelihoods, leading to conflict. In general, fisheries operate under a system of open access to fish. It has been difficult for resident communities to exclude commercial fishermen from their traditional fishing territory. When profit is the goal, they tend to catch as many fish as possible. Capture and transport of commercial fish across the Amazonian floodplain is still mainly done by fisherman’s canoe, which is evidence of artisanal fishery. The fishing boat has its role in storing fish in ice-boxes and transporting the fish caught by artisanal fishermen to the consumer centers. Fish are the main source of protein for much of the population in the Amazon River basin, exceeding 400 g of fish per person per day. Fishing is one of the most traditional activities for the region, with high socioeconomic importance. It is a source of food and income, as well as employment and leisure, for a large number of people (Batista et al. 2012).

Our fieldwork detected overexploitation of fishery resources and consequent decline in fisheries (Table 4). One piece of evidence that could well illustrate the trend for overfishing, with socioeconomic consequences, is the status of the “tambaqui” fish *C. macropomum*, which is widely appreciated and much sought after by the market. Fishing for “tambaqui” has been in decline due to overfishing since 1970, a time when this species was abundantly landed in the city of Manaus (Batista et al. 2012). The same trend occurs in relation to the other well-known Amazonian fish such as the giant “pirarucu” *Arapaima gigans*, “tucunaré” *Cichla* spp., or “jaraqui” *Prochilodus brama*, and also to regional freshwater turtles such as *Podocnemis expansa* and *P. unifilis* (Alho and Pádua 1982, Alho 1985, 2011).

The environmental threats identified in this study have strong links with fish ecology, negatively impacting their biological cycles. Amazon fish food originates in the synthesis of two sources: an autochthonous source, which depends on the productivity of the aquatic ecosystem itself (phytoplankton, zooplankton, aquatic macro-invertebrates, insect larvae, etc.) and an allochthonous source, which depends on the features near water, mainly the alluvial floodplain forest (fruit and seeds fallen into the water, etc.).

Water chemistry imposes additional constraints on distribution and abundance of Amazonian biodiversity. Direct contamination of water, as a result of the identified threat from environmental contaminants, alters fish abundance and modifies ecological community. Within the Amazon basin, there are rivers with water of different colors, including three major types: (1) Rivers which have their headwaters in uplands such as the Amazon River with its springs on the Andes, sediment-rich water with nutrients from rocky terrains. This is also the case of the Juruá, Purus, Madeira, Marañón, Meta, Napo, and Ucayali rivers. (2) Rivers with black waters due to natural dissolved organic matter such as tannins, including the Negro, Trombetas, and Tefé rivers, with very acidic waters that are nearly devoid of sediments and nutrients. These rivers rise in the Amazon forest and carry forest sediments, mainly decomposing fragments of leaves and other organic matter. (3) Rivers with clear water, relatively nutrient-poor that can range from alkaline to acidic waters, such as the Tapajós and Xingu that run throughout areas of low inundation, mainly the crystalline soils of the central Brazilian plateau, covered by savannah vegetation (Cerrado biome), before reaching the lowland seasonally flooded Amazonian “várzea.” The Upper Xingu River suffers from serious problems with deforestation, sport fishing decline, and the threat of soybean fields, and this area suffers from the intensive use of fertilizers and pesticides, contaminating the freshwater wildlife.

The scientific information available encapsulates our understanding of how the Amazon biome operates as an ecological system and how the biological resources, including fishery resources, are regulated. The highly diverse range of Amazonian biodiversity lives in a number of different aquatic habitats, including large rivers, small streams, extensive wetlands and seasonally flooded areas, rivers and lakes in elevated areas, etc. These main types of aquatic habitats are based on altitude, land gradient, rainfall, temperature, vegetation cover, and soil type. The large river channels are of immense dimensions in the Amazon, such as the Amazon River itself or the Negro River near the city of Manaus. They have sinuous courses, forming islands, and flood large portions of the forest forming the “várzea” and “igapó” seasonally inundated ecosystems.

It has been recognized that conflicts over biodiversity resources can be diminished and resources better managed when stakeholders are more involved in management, and when access rights are distributed more effectively and equitably (Ruffino 2004, 2005, 2008; Batista et al. 2012).

The suggestion to build a management strategy for Amazon fisheries resources should include an integrated bank of information, with analysis of existing data on fish biology and fisheries. It has to consider options for planning, consultation, decision-making, distribution of tasks and resources, and formulation and implementation of rules for managing all fishing activities. This management aims to ensure environmental sustainability, the continued productivity of fisheries resources, and the socioeconomic compatibility of the actors involved.

Besides the integrated approach among all actors involved in fisheries, management should also emphasize conservation of the aquatic ecosystems, considering the environmental threats analyzed in this study.

Several authors studying fisheries in the Amazon indicate some models of participatory community management of fisheries as one of the successful strategies to achieve sustainable use (Ruffino 2004, 2005, 2008; Batista et al. 2012). Artisanal fishing is still crucial for riverine populations in the Amazon. On the other hand, the increased demand for fisheries has created conflicts between commercial fishermen and the inhabitants of rivers, streams and lakes who attempt to prohibit the entry of commercial fishermen in their traditional fishing areas, faced with the new situation of declining fish stocks.

## Conclusion

In conclusion, this study shows that the conservation and management of the Amazonian aquatic ecosystems and their fishery resources can be enhanced if there is governmental and political motivation to implement management through participatory activities, integrating all actors involved, to enforce legislation and specific fishery norms. This is necessary to put into practice the laws and regulations, fishing calendar, environmental education, to minimize fishing conflicts, and to develop specific monitoring programs to mitigate the environmental threats to aquatic ecosystems. The lessons learned with some successful Amazon fishery projects are a positive direction to be taken and improved on, considering this new information on conservation and management of Amazonian freshwater ecosystems.

Sustainable fishing strives for the balance between the losses caused by human and natural growth, and the gains due to reproduction and growth, in such a way that the abundance of a species in a given area remains constant through time. Conservation and management are tools to encompass ecosystem interconnections, linking human use such as fisheries to scientific knowledge.

The efforts of Amazon human communities to reassume control of lakes and other traditional fishing territories have added the participation of riverine people, fishermen’s associations, fishing associations, governmental and non-governmental organizations, religious organizations, municipalities, and other actors involved. The idea is to implement measures to regulate fishing in certain areas, by means of a fishing agreement (acordo de pesca), for the socioeconomic benefit of local people. Consistent with this line of action, some successful projects have supported some fishing agreements in the Brazilian Amazon, and the “ProVárzea” Project has played an important role with government support. The Mamirauá Institute, located near the city of Tefé, state of Amazonas, Brazil, has been implementing participatory community-based management of “pirarucu” (*Arapaima gigas*) in the Mamirauá Reserve of Sustainable Development for over a decade, successfully both in the application of the management model and with respect to socioeconomic aspects to increase the income of local fishermen. Fishermen involved in this program recognize that resource conflicts can be diminished and better managed when they and other stakeholders are more committed to management, and when access rights are distributed more efficiently and equitably. There has been a commitment from the Brazilian government (Ibama Institute, through the “ProVárzea” program) to implement policies regarding decentralization and community-based management. Also the Fishing Colonies and Fishing Zones are organizations that can better address the relationship of fisheries resources to local fishermen’s welfare and the conservation of the resources by implementing an agreed management plan, including all the relevant attributes of legislation.
